# Response to comments on the management and ethical implications of genetic testing in CADASIL

**DOI:** 10.1055/s-0043-1777067

**Published:** 2023-11-30

**Authors:** Renata Nogueira, Christian Marques Couto, Pérola de Oliveira, Bernardo José Alvez Ferreira Martins, Vinicius Viana Abreu Montanaro

**Affiliations:** 1 Rede Sarah de Hospitais de Reabilitação, Rio de Janeiro RJ, Brazil.; 2 Rede Sarah de Hospitais de Reabilitação, Brasília DF, Brazil.

Dear Editor,

We would like to extend our gratitude to Dr. Ruiz et al. 1 for their insightful comments regarding our manuscript.

We concur that proper management of patients with hereditary cerebral small vessel disease, specifically CADASIL, should take precedence over predictive genetic testing of family members. 2

Regrettably, there are no effective pharmacological treatments available to delay or halt the progression of this disease, as well as many other late-onset neurodegenerative diseases, even with early diagnosis. Nevertheless, potential benefits of performing genetic testing on asymptomatic family members include enhanced psychosocial well-being and increased control over life decisions, such as the option for reproductive testing. 3

For ethical considerations, both genetic counseling and presymptomatic tests ought to be conducted in accordance with specific guidelines and programs to mitigate negative outcomes—what are often referred to as “catastrophic events”—such as suicide attempts or psychiatric hospitalizations. 3

Unfortunately, these guidelines are not consistently applied in practice, leading to variations in genetic counseling and testing procedures among healthcare providers. These variations can include differing roles for healthcare team members, the number and type of appointments, and specific requirements for neurological or psychiatric/psychological assessments. 4

The literature on genetic counseling for CADASIL and other cerebrovascular diseases is limited. Thus, we recommend adhering to protocols developed for conditions like Huntington's disease. 2

In Brazil, guidelines for genetic counseling in late-onset hereditary neurological disorders are scarce and only available in a few specialized services. Additionally, there remains a need for more research concerning the influence of cultural background on the acceptance of presymptomatic tests in low- and middle-income countries. 5

Our study was conducted at an international neurological rehabilitation center, which primarily serves symptomatic patients to minimize disability and enhancing functionality.

In the context of the Brazilian Public Health System (SUS), genetic counseling is primarily offered in university hospitals. Despite advances in medical genetic care, the current public services and specialized personnel in medical and human genetics fall substantially short of the country's needs. It is worth noting that molecular diagnostic technologies were not added to the list of procedures for the SUS until 2014, and even then, only rare diseases were included. 6

In conclusion, we acknowledge the importance of genetic counseling and advocate for increased efforts to broaden access to genetic tests for family members, while rigorously adhering to ethical guidelines.
